# Intercropping with faba bean under appropriate row configuration increases root yield and active ingredient content of *Codonopsis pilosula*

**DOI:** 10.3389/fpls.2025.1588096

**Published:** 2025-06-02

**Authors:** Gaojian Huang, Xingyu Qin, Ziyu Chen, Huifeng Miao, Zhiping Yang, Qiang Zhang, Yi Xing

**Affiliations:** ^1^ College of Resource & Environment, Shanxi Agricultural University, Taiyuan, Shanxi, China; ^2^ Soil Health Laboratory in Shanxi Province, Taiyuan, Shanxi, China

**Keywords:** traditional Chinese medicine, crop diversity, aboveground interaction, light interception, light use efficiency

## Abstract

Continuous cultivation of the medicinal herb *Codonopsis pilosula* (*C. pilosula*) declines root yield and active ingredient content. Scientific and reasonable intercropping patterns can improve yield and active ingredient accumulation. However, how intercropping systems affect root yield and active ingredient content of *C.pilosula* is still poorly understood. We conducted a field experiment with four treatments, including monoculture *C. pilosula* (MC), one row of *C. pilosula* intercropping with one row of faba bean (IC1), two rows of *C. pilosula* intercropping with one row of faba bean (IC2), and four rows of *C. pilosula* intercropping with one row of faba bean (IC3), to explore the response of leaf traits, root yield, and active ingredient content of *C. pilosula* to cropping patterns. The results showed that intercropping significantly increased the root yield of *C. pilosula* by 7.0-18.7%, lobetyolin yield by 8.6-25.2%, atractylenolide III yield by 34.2-54.0%, and syringin yield by 31.1-53.4% compared to monoculture, and the largest yield advantage occurred in IC2. The results also showed that intercropping significantly improves leaf size, net photosynthetic rate, and C metabolism enzyme activity of *C. pilosula*. Correlation analysis and partial least squares path model showed that improved root and active ingredient yield in intercropping can be attributed to enhanced leaf photosynthesis and C metabolism, indicating that appropriate row configuration in *C. pilosula*/faba bean intercropping system could increase the yield of *C. pilosula* by enhancing light use efficiency. These findings suggest that two rows of *C. pilosula* intercropping with one row of faba bean is a promising approach to establishing a high-yield and sustainable *C. pilosula* agroecosystem.

## 1 Introduction

*Codonopsis pilosula* is an important perennial medicinal herb in China, which dry roots have been widely used as traditional Chinese medicine (Committee National Pharmacopoeia, 2020). *C. pilosula* encompasses a variety of pharmacological ingredients, such as polysaccharides, polyacetylenes, flavonoids, alkaloids, terpenoids, etc. which have been used to nourish the spleen and lung, enhance organic immunity, promote gastrointestinal function, and so on (Liu et al., 2018a; Xu et al., 2025). Additionally, *C. pilosula* is also esteemed as a widely favored health food that is utilized as a dietary supplement in the concoction of soups, the fermentation of wines, and the preparation of teas (He et al., 2015; Liang et al., 2021). On this account, the market demand and cultivation area of *C. pilosula* have increased in recent years. However, due to the “geographical indication” of *C. pilosula*, long-term continuous cropping is commonly conducted by farmers, which resulting a significant decline in both yield and active ingredient contents, threatening the development of *C. pilosula* industry (Xu et al., 2025, 2024). For example, the planting area and yield of *Ludangshen*, an endemic species of *C. pilosula* planted in southeast Shanxi, China, is declined due to long-term continuous planting. Therefore, it is urgent to improve cultivation patterns to promote yield and active ingredient contents of *C. pilosula*.

Intercropping, as a traditional cultivation practice to increase the diversity of crops, has been repeatedly reported to enhance agroecosystem functioning, such as improving crop yield, year-to-year yield stability, grain quality, soil fertility, and decreasing environmental costs (Chai et al., 2021; Gui et al., 2024; Li et al., 2020a, 2023, 2024, 2021). Two preeminent mechanisms that explain the productivity advantages of intercropping are niche differentiation and interspecific facilitation, which promote the resource-use efficiency of light, nutrients, and water (Yu et al., 2022). For example, maize/peanut intercropping could increase the light interception of maize and reduce the light interception of peanut, while reducing the light use efficiency of maize and increasing the light use efficiency of peanut compared with monoculture, which improves the light use efficiency of the intercropping system (Wang et al., 2021b). Maize/legume intercropping could increase N acquisition due to enhanced N_2_ fixation by legumes and soil N content (Xing et al., 2023). Different from food crops, i.e. the harvested organ is seeds, the harvested organ of many medicinal plants is the underground parts, such as *Panax notoginseng* and *Atractylodes lancea*, which may result in different responses to intercropping. Several studies have shown that intercropping can increase root yield and active ingredient content of medicinal plants. *Panax notoginseng* intercropping with trees, e.g. *Platycladus orientalis* and *Schima wallichii Choisy*, would promote yield and saponin concentration of *Panax notoginseng* through changing soil metabolites (Wang et al., 2024). Intercropped with maize could improve root yield and volatile oil accumulation of *Atractylodes lancea* by ameliorating the rhizosphere soil microenvironment and changing the composition of volatile organic compounds (Peng et al., 2023a; Peng et al., 2022). Those studies focus more on the soil mechanisms of intercropping to increase productivity, and there is a lack of research on the plant morphology and physiology mechanisms. Our previous study showed that intercropped with faba bean in alternate rows improves root yield and active ingredient (i.e. polysaccharide and lobetyolin) concentration of *C. pilosula* (unpublished results), but the mechanisms are lacking.

The light environment in crop canopy is crucial to crop growth and productivity. Interception of photosynthetic active radiation (PAR) and assimilation of intercepted PAR by crops jointly determine light use efficiency, yield, and quality (Han et al., 2022; Liu et al., 2018b). Previous studies have shown that improved light use efficiency is one of the main reasons for increased productivity in intercropping systems (Wang et al., 2021b). On the one hand, intercropping alters the distribution and intensity of light, resulting in spatial niche differentiation, which improves the intercropping system’s light interception, such as maize/peanut and sorghum/soybean intercropping systems (Wang et al., 2021a, 2021). On the other hand, crops can change morphological and/or physiological traits, such as leaf area and plant height, to cope with the change of light environment in the intercropping systems, which changes the light interception, and further affects the light use efficiency (Yang et al., 2023; Zhang et al., 2023). In addition to light interception, intercropping influences the assimilation of intercepted PAR through the change of photosynthesis and C metabolism. For example, intercropped with soybean increases maize yield by improving the net photosynthetic rate and transferring assimilated products to seeds (Feng et al., 2024). Although yield formation is closely related to light use efficiency, few studies prove the roles of light resources in medicinal herb-based intercropping systems.

Row configuration in intercropping is an important factor affecting crop productivity because it can change the field micro-climate, especially the light conditions, and regulate the competitive relationship between intercropped species (Kou et al., 2024; Wang et al., 2021a). For example, in the maize/peanut intercropping systems, 4 rows of maize intercropped with 8 rows of peanut had the highest system yield by changing the light environment of the peanut canopy and light use efficiency, meaning a scientifically reasonable row configuration can improve light use efficiency and the yield enhancement of intercropping systems to the greatest extent (Lu et al., 2023). In addition, appropriate width configuration of sorghum/soybean intercropping could improve dry matter formation and N, P, K absorption in sorghum roots due to improved light environment (Peng et al., 2023b). Thus, exploring the appropriate row configuration of the *C. pilosula*/faba bean intercropping system is a meaningful step to increase the root yield and active ingredient concentration of *C. pilosula*.

In this study, we conducted a field experiment to explore the effects of row configuration on the leaf morphological trait (i.e. leaf size), physiological characteristics (i.e. photosynthesis, C and N metabolism), root yield, and active ingredients accumulation of *C. pilosula* in *C. pilosula*/faba bean intercropping system. We hypothesized that proper row configuration could improve root yield and active ingredient accumulation of *C. pilosula* by improving leaf morphological and physiological traits. Our objectives are to: (1) investigate the response of leaf morphology and physiology traits, root yield, and active ingredient content of *C. pilosula* to row configurations; (2) explore how the changes in leaf morphology and physiology under different row configurations affect the root yield and active ingredient content of *C. pilosula*; (3) identify the appropriate row configuration for the highest yield and active ingredient content.

## 2 Materials and methods

### 2.1 Site description

The field experiment was conducted in 2022 at Shijiapo Village, Jincheng City, Shanxi Province (35˚79′N, 113˚45′E, and altitude 1200 m). The region is characterized by a typical temperate continental climate with a mean annual precipitation of 650 mm and a mean annual temperature of 7.9 ˚C. The soil is classified as Cinnamon Soil (WRB, 2015). The soil (0–20 cm) before plant sowing had an organic matter concentration of 28.72 g kg^-1^, total nitrogen concentration of 2.06 g kg^-1^, available nitrogen concentration of 32.80 mg ka^-1^, available phosphorous concentration of 13.99 mg ka^-1^, available potassium concentration of 206.55 mg kg^-1^.

### 2.2 Plant material

*Ludangshen*, a *C. pilosula* variety from Changzhi City, Shanxi Province, China, was planted in the present study. *Ludangshen* presents a specific root characteristic with root head like a “lion’s head”, root tail like a “phoenix’s tail”, and root cross-section like a “chrysanthemum”. Continuous planting of *Ludangshen* in Changzhi City has resulted in the loss of root yield and the reduction of active ingredient content. Previous studies have focused more on the pharmacology and nutrient management of *Ludangshen*, with little attention to its cultivation.

### 2.3 Experimental design

**Figure 1:**
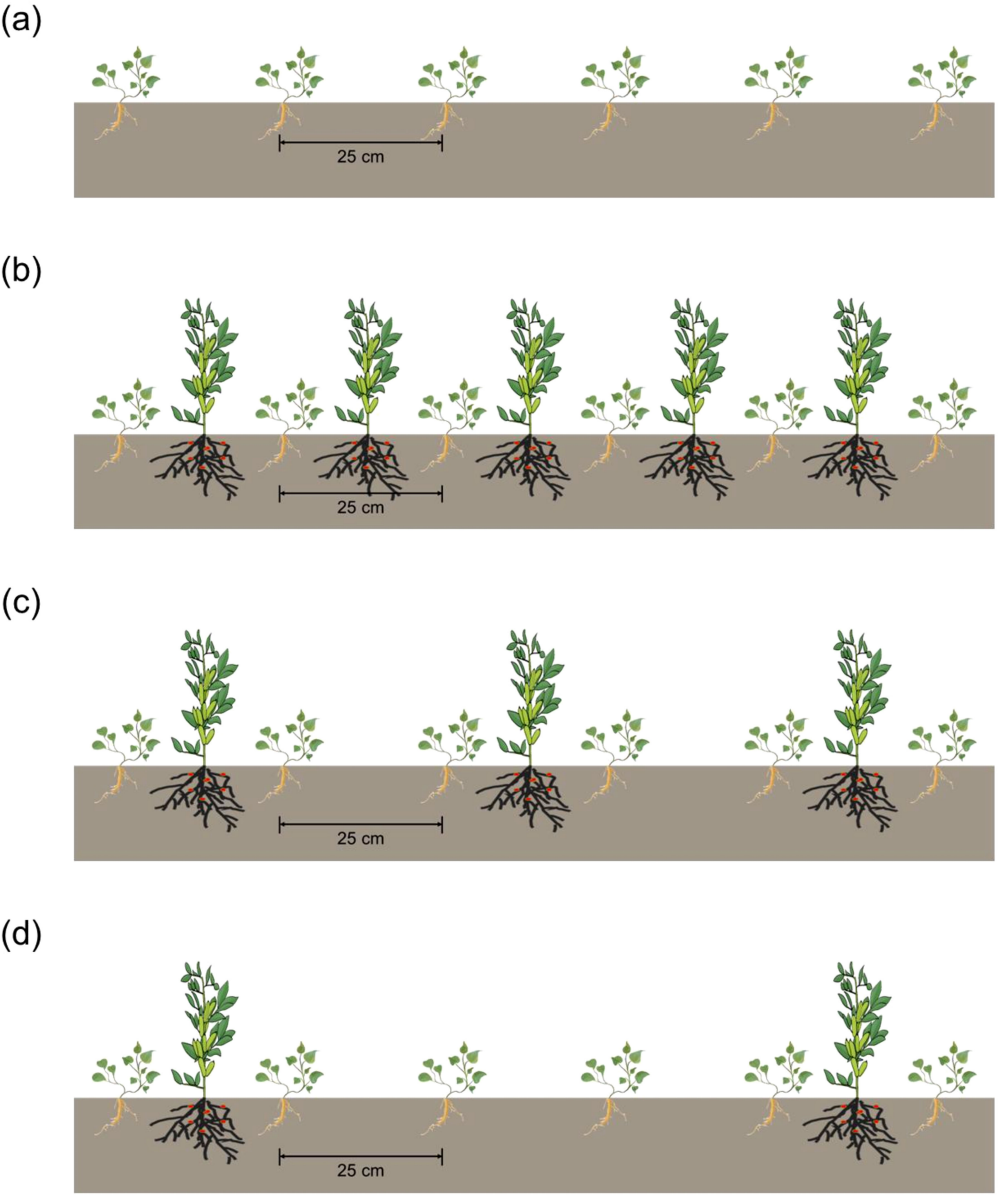
Schematic diagram of *C. pilosula*/faba bean intercropping and monoculture treatments. **(a)** monoculture *C. pilosula*; **(b)** one row of *C. pilosula* intercropping with one row of faba bean; **(c)** two rows of *C. pilosula* intercropping with one row of faba bean; **(d)** four row of *C. pilosula* intercropping with one row of faba bean.

**Figure 2:**
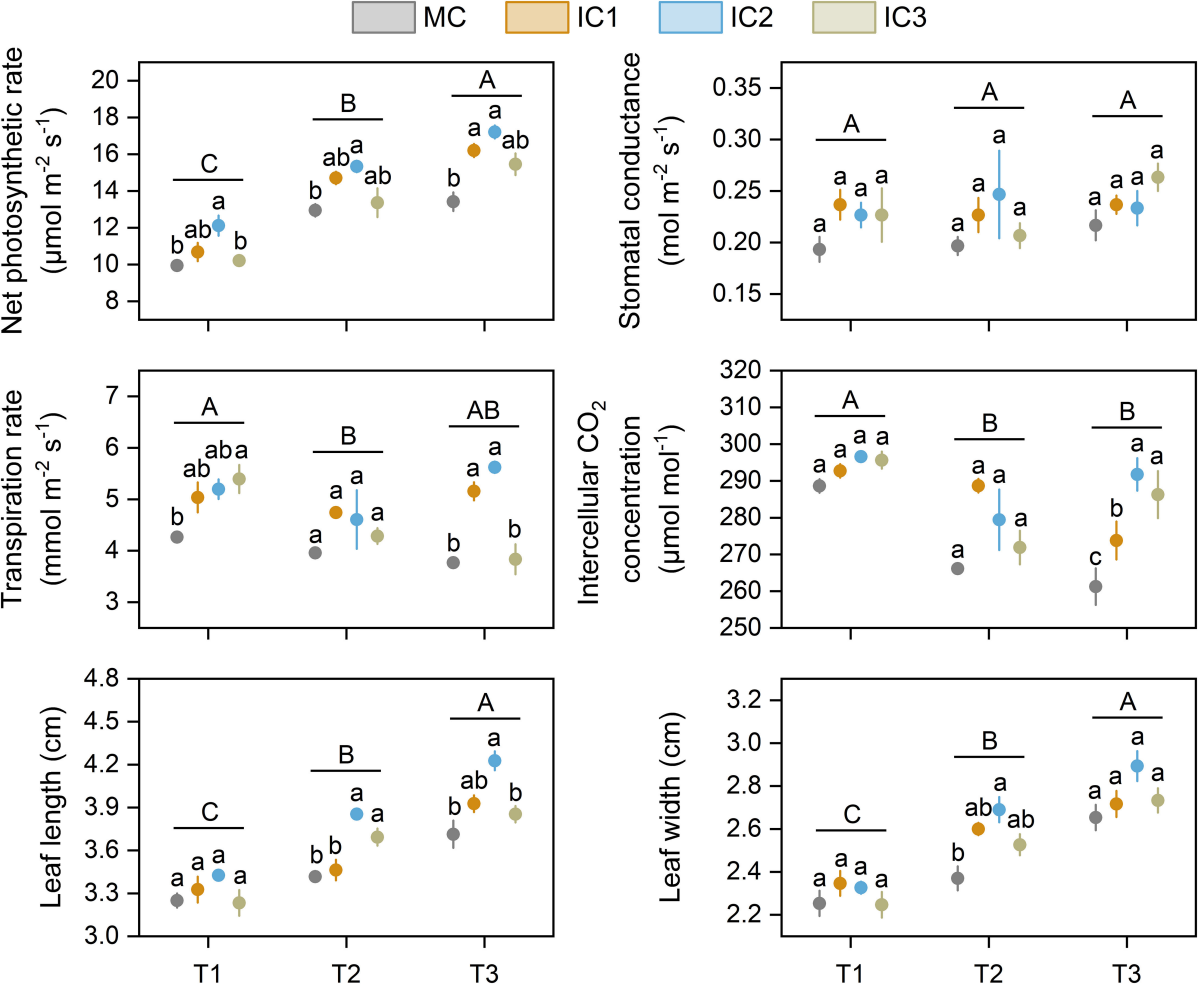
Leaf photosynthetic parameters and size of *C. pilosula* at different stages. Means and standard error are presented (n = 3). Different lowercase letters indicate significant differences among treatments at same growth stage, and different capital letters indicate significant differences among growth stages (*P* < 0.05, Tukey HSD). T1: seedling stage, T2: early flowering stage, T3: peak flowering stage.

**Figure 3:**
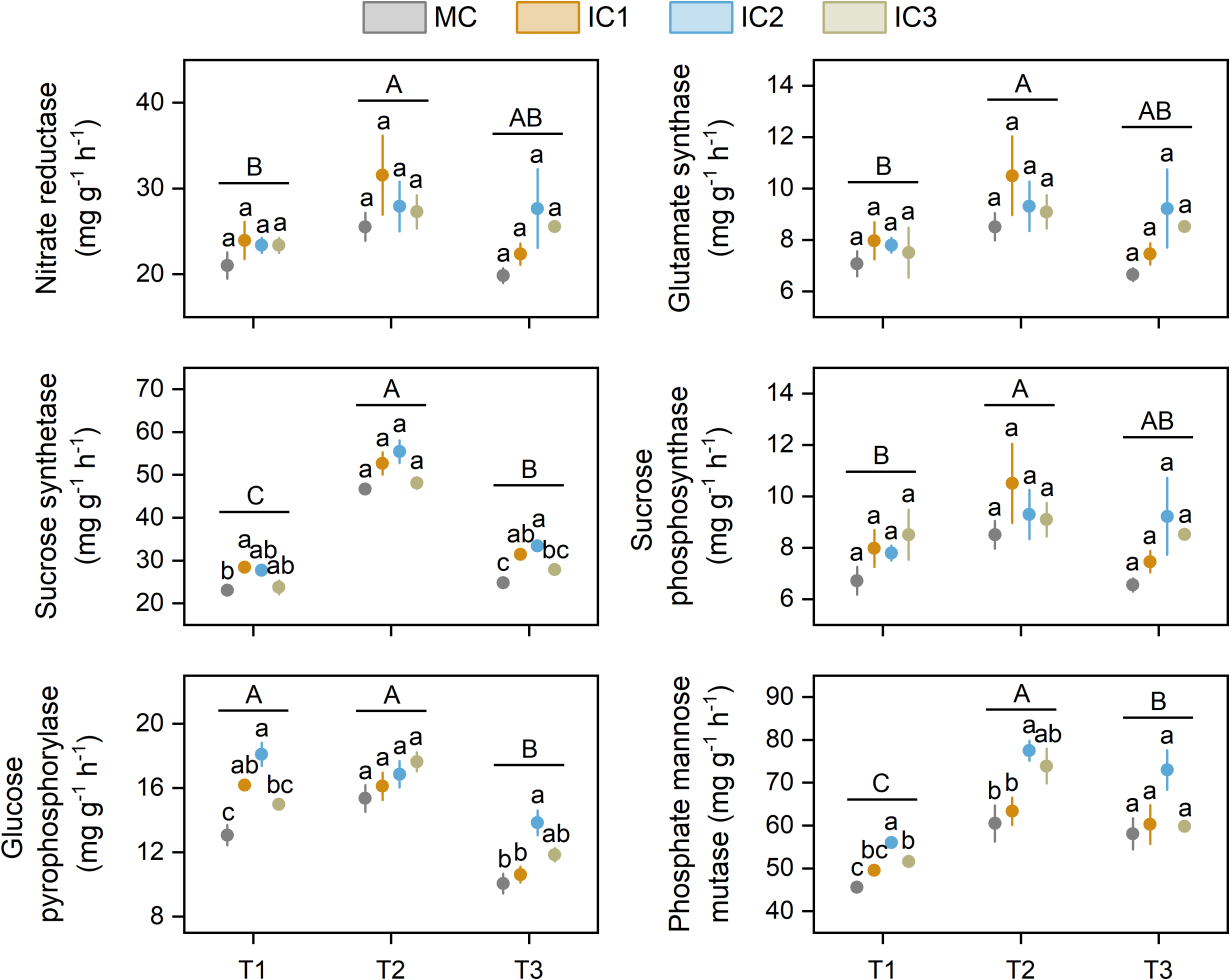
Nitrogen and carbon metabolism enzyme activities of *C. pilosula* leaf at different stages. Means and standard error are presented (n = 3). Different lowercase letters indicate significant differences among treatments at same growth stage, and different capital letters indicate significant differences among growth stages (*P* < 0.05, Tukey HSD). T1: seedling stage, T2: early flowering stage, T3: peak flowering stage.

The field experiment was arranged in a randomized block design with four cropping systems and three replicates. In total, there were 12 plots, with each 24 m^2^. Monoculture *C. pilosula* (MC) was planted as control, the inter-row and inter-plant distance between *C. pilosula* was 20 cm × 8 cm (**Figure 1a**). Three *C. pilosula*/faba bean (*Vicia faba* L. cv. Lincan No. 9) intercropping systems were established as follows: (1) one row of *C. pilosula* intercropping with one row of faba bean (IC1), the planting density of intercropped *C. pilosula* was the same with monoculture, and faba bean was planted with a 25 cm inter-row distance and 25 cm inter-plant distance (**Figure 1b**); (2) two rows of *C. pilosula* intercropping with one row of faba bean (IC2), faba bean was planted with a 50 cm inter-row distance and 25 cm inter-plant distance (**Figure 1c**); (3) four rows of *C. pilosula* intercropping with one row of faba bean (IC3), and faba bean was planted with a 100 cm inter-row distance and 25 cm inter-plant distance (**Figure 1d**). The *C. pilosula* and faba bean were planted on 23 March and 6 May, separately, and harvested on 15 October.

All plots were given identical applications of 60 kg N ha^-1^ as urea, 46 kg P ha^-1^ as superphosphate, and 54 kg K ha^-1^ as potassium sulfate. Before sowing, P, K, and one-half of N fertilizer were evenly broadcast and incorporated into the soil, and the other half of N fertilizer was applied at the pre-flowering stage of the *C. pilosula* (27 July). The study site is situated in a rain-fed agriculture region with a mean annual precipitation of 650 mm, which can meet the water needs of *C. pilosula* growth. Thus, no irrigation was applied during the growing period of *C. pilosula*, according to the practice of local farmers. All plots were weeded manually.

### 2.4 Plant sampling and analysis

Plant sampling was conducted at the seedling stage (27 June), early flowering stage (22 July), peak flowering stage (3 September), and maturing stage to define the effects of intercropping patterns on *C. pilosula*.

Leaf photosynthetic parameters of *C. pilosula*, i.e. net photosynthetic rate, stomatal conductance, transpiration rate, and intercellular CO_2_ concentration, were measured using a Li-6400X system (LI-COR, Lincon, NE, USA) with a constant CO_2_ concentration of 400 μmol mol^-1^ between 9:00-11:00 a.m. Photosynthetic parameters were measured within each plot for six plants, the first fully expanded leaf for each plant was taken as the material for the determination. In the IC3 treatment, photosynthetic parameters of *C. pilosula* were measured in both border and inner rows.

Leaf and root of *C. pilosula* were sampled at the seedling, early flowering, and peak flowering stages. Six plants with uniform growth in each plot were taken to collect mature leaves and roots. Leaves were divided into two parts, one for measuring leaf length and width, and the other was stored in liquid nitrogen for measuring enzyme activity. Roots were sampled by digging 25 cm wide and 30 cm deep with a shovel, and roots were washed carefully to remove the remaining soil, and then root length and diameter were measured by ruler and vernier caliper, separately. Subsequently, roots were over-dried at 105 °C for 30 min and 70 °C for 72 h and then weighted to determine dry weight. Dry roots were ground to a fine powder and used for metabolite concentration analyses. At harvest, Root fresh yield was determined by harvesting 4 m^2^ *C. pilosula* in each plot.

Sucrose synthetase (SS), sucrose phosphosynthase (SPS), glucose pyrophosphorylase (UGPase), and phosphate mannose mutase (PMM) were assayed in frozen leaf tissue to reflect the level of carbon metabolism in *C. pilosula*. Nitrate reductase (NR) and glutamate synthase (GOGAT) were assayed in frozen leaf tissue to reflect the level of nitrogen metabolism in *C. pilosula*. The activity levels of SS, SPS, UGPase, PMM, NR, and GOGAT were measured by the sucrose synthetase assay kit (Solarbio, Beijing, China), the sucrose phosphoric acid synthetase assay kit (Solarbio, Beijing, China), the UDP-glucose pyrophosphorylase assay kit (Bohu, Shanghai, China), the phosphate mannose mutase assay kit (Bohu, Shanghai, China), the nitrate reductase assay kit (Solarbio, Beijing, China), and the glutamate synthase assay kit (Solarbio, Beijing, China), respectively, according to the manufacturer’s instructions. Each sample have two parallel tests.

Lobetyolin, atractylenolide III, and syringin concentration were determined using the method described by Huang et al. (2024). Briefly, 0.1 g root sample powder was extracted with 25 mL 75% methanol for 45 min at 40 °C by ultrasonication (50 kHz, 40 W). After centrifugation at 10000 r min^-1^ for 15 min, the supernatant was filtered through a 0.22-μm membrane. Filtered samples were analyzed by an ACQUITY UPLC BEH C18 column (2.1 mm × 50 mm, 1.7 μm; Waters Corporation, USA). The column temperature was 30 °C, the flow rate was 0.4 mL min^-1^, and the injection volume was 2 μL. Injections were gradient-eluted using a mobile phase consisting of methanol (A) and 0.1% ammonium formate solution (B) with the following gradient conditions: 5% A at 0-0.5 min; 5-95% A at 0.5–5 min; 95% A at 5–7 min; 95%-5% A at 7-7.1 min; 5% A at 7.1–9 min, and the detection wavelength was 220 nm. Standard lobetyolin, atractylenolide III, and syringin were obtained from the China National Institutes for Drug Control, and the standard dilutions were 10, 50, 100, 400, 600, and 1000 ng mL^-1^. Each sample have two parallel tests.

### 2.5 Statistical analysis

Two-way analysis of variance (ANOVA) was conducted to evaluate the effects of growth stage, cropping system, and their interaction on leaf size, photosynthetic characteristics, C and N metabolism enzyme activity, root properties, and active ingredient concentration by using linear mixed model with growth stage and cropping system as fixed effects and repeat as random effect. One-way ANOVA was conducted to test the effects of cropping system on root yield and active ingredient yield by using linear mixed models with cropping system as a fixed effect and repeat as random effects. Multiple comparisons were determined by Tukey’s HSD test at *P* < 0.05. Pearson correlation was conducted to evaluate the relationships between leaf traits and yield. A principal component analysis (PCA) among leaf and root traits was conducted using *vegan* package. A partial least squares-path analysis (PLS-PM) in R package *plspm* was used to explore relationships among leaf traits, root yield, and active ingredient content. All statistical analyses were conducted with R version 4.1.3 (R Development Core Team, 2022).

## 3 Results

### 3.1 Leaf photosynthetic parameters and leaf size

Net photosynthetic rate increased with growth stage, while intercellular CO_2_ concentration decreased with growth stage. The effects of cropping systems on leaf photosynthetic parameters depended on the growth stages of *C. pilosula*. Compared with monoculture, intercropping increased net photosynthetic rate by 2.7%-21.9% at the seedling stage (*P* < 0.05), 3.2%-18.5% at early flowering stage (*P* < 0.05), and 15.2%-28.2% at peak flowering stage (*P* < 0.01), respectively (**Figure 2**). The increased effect was more pronounced at IC2 than at other treatments. Intercropping did not affect the leaf stomatal conductance of *C. pilosula* across the growth stages. Intercropping significantly increased the leaf transpiration rate by 18.1%-26.5% at the seedling stage (*P* < 0.05) and 1.8%-49.3% at the peaking flowering stage (*P* < 0.001) compared with monoculture but did not affect it at the early flowering stage (*P* = 0.33). Compared with monoculture, intercropping significantly increased intercellular CO_2_ concentration by 4.8%-11.7% at the peak flowering stage (*P* < 0.001), but did not at the seedling (*P* = 0.08) and early flowering stage (*P* = 0.06). There were no significant differences in leaf instantaneous water use efficiency among cropping systems at the same stage (**Table 1**).

**Table 1:** Effects of different cropping systems on leaf instantaneous water use efficiency at different growth stages.

Treatments	T1	T2	T3
MC	2.33 ± 0.06bcd	3.27 ± 0.11ab	3.57 ± 0.18a
IC1	2.13 ± 0.08cd	3.10 ± 0.06abc	3.15 ± 0.10abc
IC2	2.35 ± 0.19bcd	3.45 ± 0.51a	3.06 ± 0.02abc
IC3	1.91 ± 0.12d	3.14 ± 0.27abc	4.06 ± 0.19a

Means and standard error are presented (n = 3). Different lowercase letters indicate significant differences among cropping systems at different stages (*P* < 0.05, Tukey HSD).

Leaf length and width increased with growth stage. Intercropping increased leaf length at the early (*P* < 0.01) and peak (*P* < 0.05) flowering stages (**Figure 2**). IC2 treatment had the largest increase with 12.8% higher at the early flowering stage and 13.8% higher at the peak flowering stage than monoculture. Compared with monoculture, intercropping significantly increased leaf width by 6.6%-13.5% at the early flowering stage (*P* < 0.05), but not at the seedling and peak flowering stages (**Figure 2**).

### 3.2 Leaf metabolism enzyme activities

The largest enzyme activities were found at the early flowering stage (**Figure 3**). Intercropping did not affect nitrate reductase and glutamate synthase activity across the three growth stages.

The cropping system significantly affected the activity of sucrose synthetase, glucose pyrophosphorylase, and phosphate mannose mutase (**Figure 3**). Compared with monoculture, intercropping significantly increased sucrose synthetase activity by 3.1%-23.3% at the seedling stage (*P* < 0.05) and 12.4%-34.5% at the peak flowering stage (*P* < 0.01). Intercropping did not affect sucrose phosphosynthase activity across the growth stage. The activity of glucose pyrophosphorylase in intercropping was 14.6%-38.5% and 5.5%-37.7% higher at the seedling (*P* < 0.01) and early flowering stage (*P* < 0.05) than in monoculture. Intercropping significantly increased phosphate mannose mutase activity by 8.7%-23.0% at the seedling stage (*P* < 0.01), 4.7%-28.1% at the early flowering stage (*P* < 0.05), and 2.9%-25.7% at the peak flowering stage (*P* = 0.05).

### 3.3 Root properties and yield

**Figure 4:**
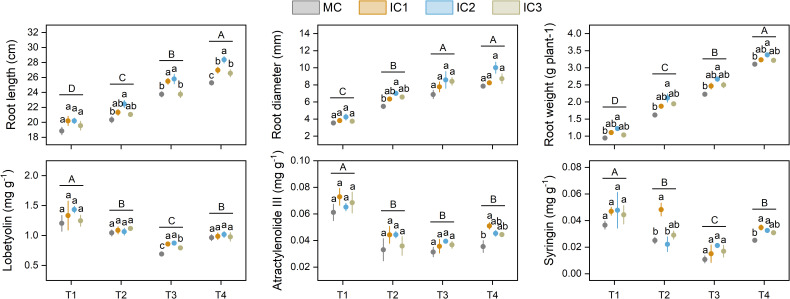
Root properties and active ingredient concentration of *C. pilosula* at different stages. Means and standard error are presented (n = 3). Different lowercase letters indicate significant differences among treatments at same growth stage, and different capital letters indicate significant differences among growth stages (*P* < 0.05, Tukey HSD). T1: seedling stage, T2: early flowering stage, T3: peak flowering stage, T4: harvesting stage.

Root length, root diameter, and single root weight increased with growth stage. Intercropping increased root length, root diameter, and single root weight, meanwhile, the increased effect was more pronounced at IC2 than at other treatments (**Figure 4**). Specifically, intercropping significantly increased root length by 3.6%-10.5% at the early flowering stage (*P* < 0.05), 7.3%-8.6% at the peak flowering stage (*P* < 0.05), and 5.1%-12.2% at the harvesting stage (*P* < 0.001). Root diameter in the intercropping system was significantly higher than in monoculture at the early flowering stage, but not significant at other growth stages. Compared with monoculture, intercropping increased single root weight by 9.2%-29.2% at the seedling stage (*P* < 0.05), 16.1%-30.9% at the early flowering stage (*P* < 0.05), 10.9%-19.9% at the peak flowering stage (*P* < 0.05), and 3.7%-8.8% at the harvesting stage (*P* < 0.05).

Overall, the active ingredient concentration decreased from seedling stage to peaking flowering stage, and then increased at harvesting. The effect of intercropping on the active ingredient concentration of *C. pilosula* was different at different growth stages (**Figure 4**). Compared with monoculture, intercropping significantly increased lobetyolin concentration by 15.0%-26.6% at the peak flowering stage (*P* < 0.001), but no effect at other growth stages. Intercropping only increased atractylenolide III and syringin concentration at the harvesting stage.

**Figure 5:**
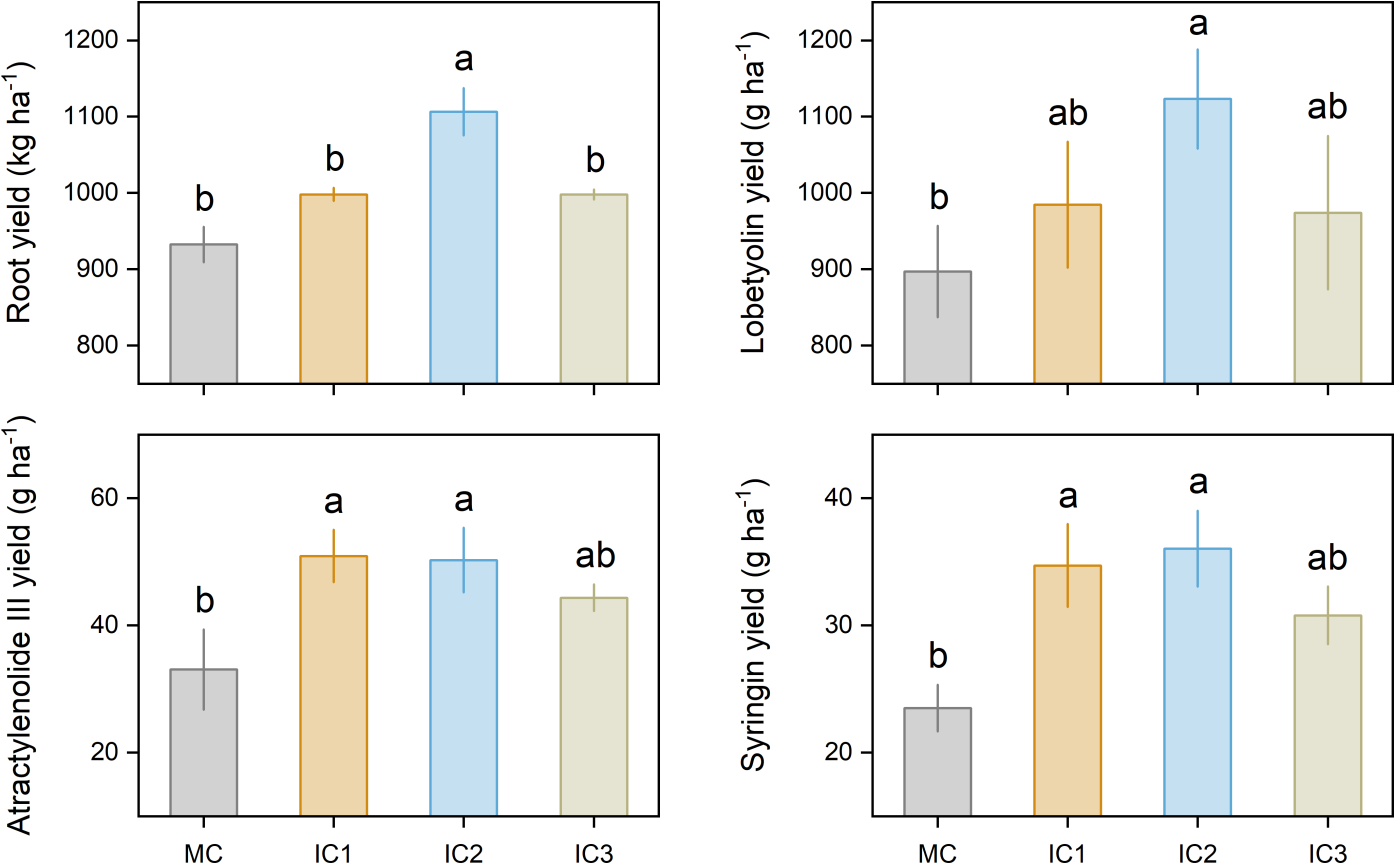
Root yield and active ingredient yield of *C. pilosula*. Means and standard error are presented (n = 3). Different lowercase letters indicate significant differences among cropping systems (*P* < 0.05, Tukey HSD).

Intercropping significantly increased root yield and active ingredient accumulation (**Figure 5**). IC1, IC2, and IC3 treatments increased root yield by 7.1%, 18.7%, and 7.0% compared to monoculture, respectively. Compared with monoculture, IC1, IC2, and IC3 treatments increased lobetyolin yield by 9.7%, 25.2%, and 8.6%, atractylenolide III yield by 54.0%, 52.0%, and 34.2%, syringin yield by 47.8%, 53.4%, and 31.1%.

### 3.4 Relationships among leaf morphological and physiological traits, active ingredient concentration, and root yield

**Figure 6:**
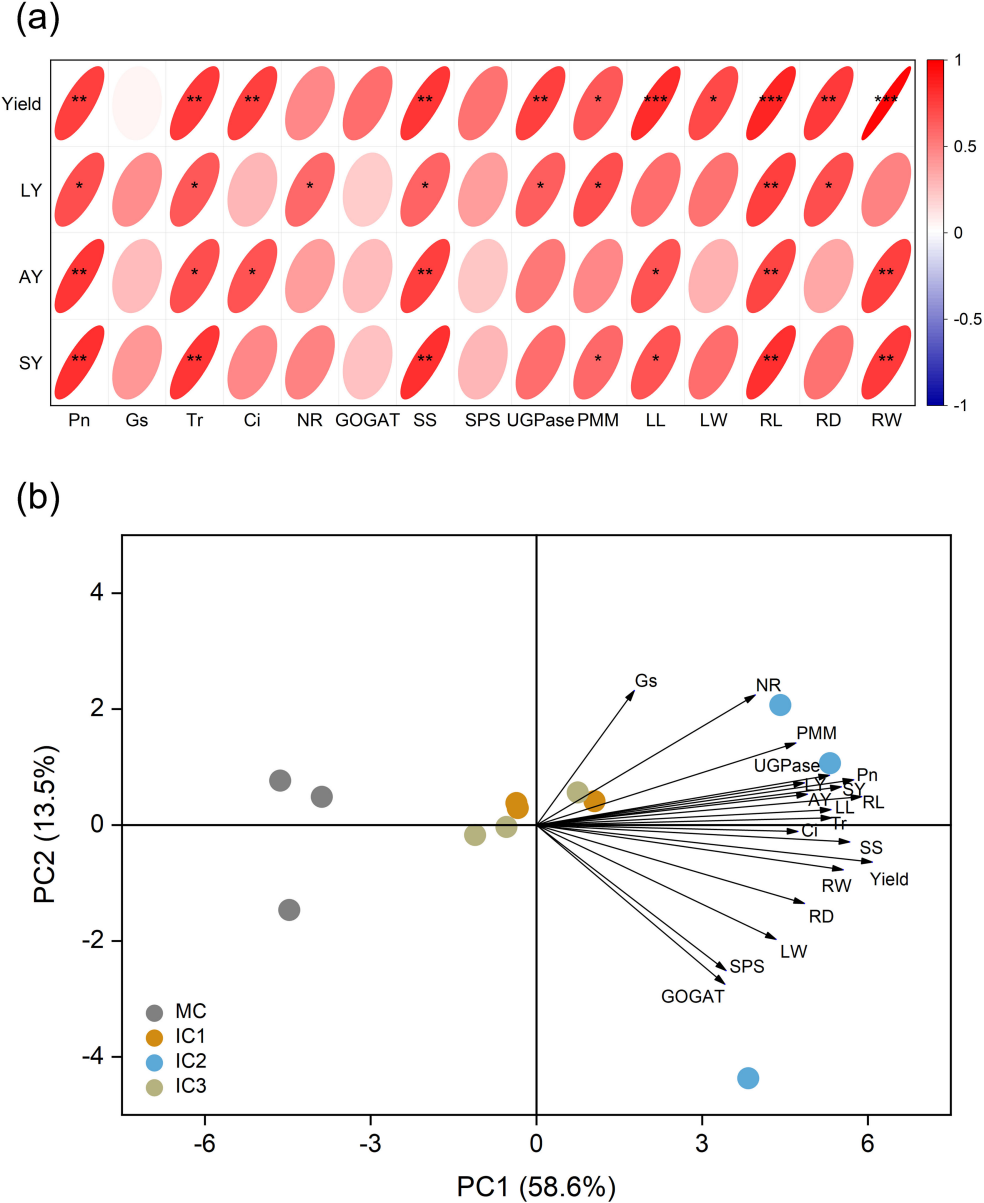
**(a)** Pearson’s correlation heatmap. The color and size of the ellipse chart represent the magnitude and direction of the relationship, respectively. *P < 0.05; **P < 0.01; ***P < 0.001. **(b)** Principal component analysis of root yield, active ingredient yield, photosynthesis, enzyme activity, and root property of *C. pilosula*. The percentage number represents proportion of variation for which the axis accounts. Pn, Net photosynthetic rate; Gs, Stomatal conductance; Tr, Transpiration rate; Ci, Intercellular CO_2_ concentration; NR, Nitrate reductase; GOGAT, Glutamate synthase; SS, Sucrose synthetase; SPS, Sucrose phosphosynthase; UGPase, Glucose pyophosphorylase; PMM, Phosphate mannose mutase; LL, Leaf length; LW, Leaf width; RL, Root length; RD, Root diameter; RW, Single root weight; LY, Lobetyolin yield; AY, Atractylenolide III yield; SY, Syringin yield.

Pearson correlation analysis revealed that root yield, lobetyolin yield, atractylenolide III yield, and syringin yield were strongly associated with leaf net photosynthetic rate, transpiration rate, sucrose synthetase, leaf length, and root length (**Figure 6a**). Principal component analysis (PCA) discriminated different cropping patterns on the PC1 axis, which explained 58.6% of the total variation (**Figure 6b**). Meanwhile, IC2 was positioned with higher net photosynthetic rate, leaf length, sucrose synthetase, root length, root yield, and metabolite yield than other cropping patterns.

**Figure 7:**
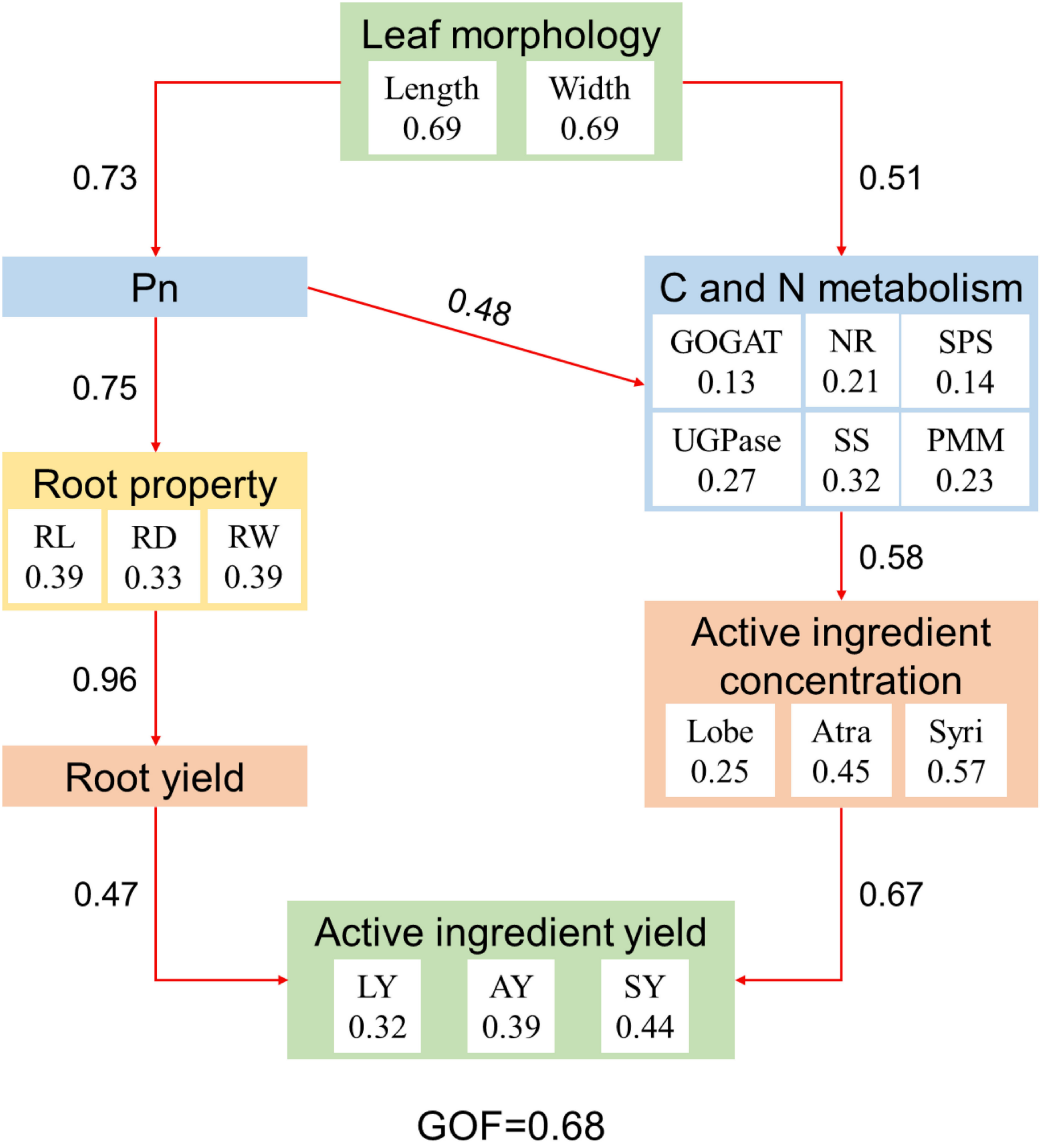
Partial least squares path model (PLS-PM) showing the relationships among leaf morphology, photosynthesis, C and N metabolism, root property, active ingredient concentration, root yield, and active ingredient yield. The goodness of fit (GOF) was 0.68, which was larger than the 0.36 cutoff. Red arrows indicate positive effect. The numbers within arrows are the standardized path coefficient. Pn, Net photosynthetic rate; NR, Nitrate reductase; GOGAT, Glutamate synthase; SS, Sucrose synthetase; SPS, Sucrose phosphosynthase; UGPase, Glucose pyophosphorylase; PMM, Phosphate mannose mutase; RL, Root length; RD, Root diameter; RW, Single root weight; Lobe, Lobetyolin; Atra, Atractylenolide III; SY, Syringin; LY, Lobetyolin yield; AY, Atractylenolide III yield; SY, Syringin yield.

Partial least squares path model (PLS-PM) analysis showed that leaf morphology (i.e. leaf length and width) of *C. pilosula* had a positive effect on active ingredient yield through induced changes in leaf photosynthesis and C/N metabolism enzyme activities, which promoted the root yield and active ingredient concentration (**Figure 7**). Leaf C and N metabolism directly influence active ingredient concentration, and leaf photosynthesis indirectly and positively affected active ingredient concentration. Active ingredient concentration was crucial for active ingredient yield (total effect = 0.67).

## 4 Discussion

### 4.1 Intercropping changes the leaf size and photosynthetic characteristics of *C. pilosula*

Light environment and radiation use are important factors determining the synthesis and accumulation of photosynthetic products of crops, which directly affects crop growth, yield, and quality (Li et al., 2021a; Lu et al., 2023; Wang et al., 2021a). An intercropping system can change the light environment and radiation use efficiency by arranging crop combinations, row configurations, and crop density.

The leaf is the main organ of crops to use light energy and conduct photosynthesis, and leaf size directly determines the amount of intercepted photosynthetically active radiation. In general, a change in light conditions may induce leaf phenotypic changes in the crops, for example, soybean intercropped with maize have a greater specific leaf area than soybean monoculture due to being shaded by maize (Yang et al., 2023). Larger leaves can help crops capture more light resources to alleviate the reduction of light interception in shaded environments, as well as increase radiation use efficiency, which improves the dry matter formation and yield of plants (Wang et al., 2020; Zhang et al., 2019). In the present study, the leaf length and width of *C. pilosula* in intercropping systems were greater than that of monoculture (**Figure 2**), meaning that *C. pilosula*/faba bean intercropping system can increase the leaf area of *C. pilosula*, indicating that intercropped *C. pilosula* can intercept much light than monoculture. Moreover, this study showed that changing row configuration has a significant effect on the leaf size of *C. pilosula* in intercropping systems, i.e. leaf size of *C. pilosula* in IC2 treatment was greater than in IC1 and IC3 treatment, indicating that changing row configuration is one of the main factors that altering the light distribution, and changing the intercepted photosynthetically active radiation in *C. pilosula*/faba bean intercropping system (Raza et al., 2021).

Photosynthesis is the main pathway of carbohydrate synthesis, and carbohydrate accumulation and distribution are directly affected by photosynthetic rate (Li et al., 2023b). Changes in the light conditions received by the crops under intercropping systems have led to significant changes in the photosynthetic character of the crop (Han et al., 2022). In general, intercropping can increase the leaf photosynthetic capacity of tall-statured crops, while decreasing that of low-statured crops, due to superior light competition of the former (Wang et al., 2021b). The present study showed that *C. pilosula*/faba bean intercropping systems increase leaf photosynthetic parameters (i.e. Pn, Tr, Gs, and Ci) of *C. pilosula* at the three growth stages, indicating that intercropping increased the photosynthetic capacity of *C. pilosula*. The increments could be due to improving the light environment and reducing the shade of C.pilosule. There are two potential reasons to explain the results. First, the soft and creeping stems of *C. pilosula* play an important role when intercropped with faba bean, because they can climb and entwine with the erect growth of faba bean to capture more light. Second, biological nitrogen fixation by faba bean play an important role when intercropping with *C. pilosula*, as they can provide more nitrogen to C.pilosual through direct or indirect nitrogen transferring, leading to a higher leaf N content in *C. pilosula*, which is conducive to the synthesis, formation, and stability of chlorophyll (Li et al., 2020b; Thilakarathna et al., 2016). The leaf net photosynthetic rate of *C. pilosula* in the IC2 treatment was higher than that in the IC1 and IC3 treatments, indicating that the appropriate row configuration of *C. pilosula*/faba bean intercropping system was beneficial in alleviating the effects of the intraspecific and interspecific light competition of intercropping system on the reduction of photosynthetic rate in the leaf of *C. pilosula*. The result also showed that intercropping did not affect the leaf instantaneous water use efficiency of *C. pilosula*. This might be because precipitation could meet the demand of *C. pilosula*, and water was not a limiting factor.

### 4.2 Intercropping changes the carbon and nitrogen metabolite enzyme activity in the leaf of *C. pilosula*

Carbon metabolism enzymes, such as sucrose synthetase (SS), sucrose phosphosynthase (SPS), glucose pyrophosphorylase (UGPase), and phosphate mannose mutase (PMM) play a critical role in carbohydrates (e.g. sucrose, starch, cellulose, etc) accumulation across plant organs (Hu et al., 2015; Tung et al., 2018). SS has a dual function in sucrose synthesis and decomposition; SPS is a key enzyme involved in sucrose synthesis, which catalyzes the reaction of uridine diphosphate glucose and fructose-6-phosphate to produce sucrose-6-phosphate; UGPase catalyzes glucose-1-phosphate to form UDP-glucose, an important precursor of carbohydrate; PMM plays a significant role in mannose metabolism, which catalyzes the interconversion between mannose-1-phosphate and mannose-6-phosphate. Those enzymes are affected by various factors, such as light intensity, photosynthesis, and soil nutrients (Qi et al., 2023; Tung et al., 2018). The present study showed that *C. pilosula*/faba bean intercropping increased leaf SS, UGPase, and PMM activity of *C. pilosula* (**Figure 3**), indicating that intercropping improved the carbon metabolism of *C. pilosula*. This change could be attributed to two reasons. First, the C metabolism enzymes are sensitive to light and photosynthesis (Qi et al., 2023). The improved light environment in *C. pilosula*/faba bean intercropping systems increased leaf light intercept and photosynthesis, thus enhancing the enzyme activities, the PLS-PM also proves the effects of light on enzyme activities (**Figure 7**). Second, N can regulate carbohydrate accumulation in plant organs by activating related enzymes (Gong et al., 2022). In the *C. pilosula*/faba bean intercropping systems, biological nitrogen fixation by faba bean could provide extra N for *C. pilosula*, thereby stimulating C-related enzyme activities. These findings imply that *C. pilosula*/faba bean intercropping can increase carbon metabolism by improving the light environment and N content of the *C. pilosula* plant.

Nitrate reductase (NR) and glutamate synthase (GOGAT) are crucial enzymes for N assimilation and transformation in plants (Gong et al., 2022; Liu et al., 2021). Previous studies have found that cereal intercropped with legumes can significantly increase the N metabolism enzyme activity of cereal compared with monoculture, such as maize/soybean and maize/peanut intercropping systems (Li et al., 2022; Nasar et al., 2022). This can be attributed to the nitrogen fixation by legumes, which helps increase the N content of cereal crops, thereby improving the N metabolism enzyme activity of cereal crops. However, we found that *C. pilosula*/faba bean intercropping had no impact on NR and GOGAT activities in this study (**Figure 3**), which is in accordance with the previous finding that wheat/faba bean intercropping did not affect GOGAT activity of wheat when N fertilizer is overused (Liu et al., 2021). In other words, the role of intercropping in N metabolism enzyme activity might be determined by N levels. Hence, we speculated that the unchanged NR and GOGAT activities of intercropped *C. pilosula* can be due to the high N content in soil and sufficient N fertilizer, which offset the effect of biological N fixation by faba bean.

### 4.3 Intercropping improved root yield and active ingredient content of *C. pilosula*

Much research has shown that intercropping can improve the productivity of the intercrops due to niche partitioning and resource complementarity (Yu et al., 2022). In this study, two rows of *C. pilosula* intercropped with one row of faba bean (IC2) significantly increased the root yield of *C. pilosula*, while other intercropping treatments exerted no effect on the root yield of *C. pilosula* (**Figure 5**), indicating that *C. pilosula*/faba bean intercropping with appropriate row configuration could improve the productivity of *C. pilosula*. Similar results have been reported in other medicinal plants’ intercropping systems. For example, intercropping with an appropriate population of pigeon pea led to a significant increase in the yield of kalmegh herb (Verma et al., 2021). Similarly, *Atractylodes lancea* intercropped with maize resulted in increased rhizome yield of *A.lancea* (Peng et al., 2023a, Peng et al., 2022). Those findings suggested that the interaction between food crops and medicinal plants may play a crucial role in increasing the yield of medicinal plants. In the current study, the root yield of *C. pilosula* is positively correlated with leaf size and net photosynthesis rate, suggesting that intercropping with faba bean could improve leaf light interception as well as photosynthesis of *C. pilosula*, which is conducive to the organic matter synthesis and accumulation, as well as yield formation. The PLS-PM further confirmed this finding.

The accumulation of active ingredients in plants is an important indicator to evaluate the quality of traditional medicinal materials. The secondary metabolites, such as Lobetyolin, atractylenolide III, and syringin, are the main active ingredients of *C. pilosula*, which have certain pharmacological effects (Huang et al., 2024). Lobetyolin is a polyacetylene glycoside compounds, which was formed by lobetyol and UDP-glucose, but the biosynthesis pathway of lobetyolin remains largely uncharacterized (Xu et al., 2024). Atractylenolide III is a compound of sesquiterpene lactones, which is synthesized through the mevalonic acid pathway and catalysis of sesquiterpene synthase (Ji et al., 2019). Syringin is a phenylpropanoid glycoside compound, which is synthesized through the phenylpropane metabolic pathway and glycosidation by UDP-glucose. In this study, we found that intercropping with faba bean significantly improved the concentration of atractylenolide III and syringin in *C. pilosula*, without affecting the concentration of lobetyolin (**Figure 4**). Moreover, by combining yield data, we found that intercropping increased the accumulation of lobetyolin, atractylenolide III, and syringin per unit area of *C. pilosula*, and the enhancements were more pronounced at IC2 treatment (**Figure 5**). Those results suggested that appropriate intercropping can promote the accumulation of active ingredients in *C. pilosula*. Based on the growth-differentiation balance hypothesis, this might be due to the accumulation of primary metabolites (e.g. carbohydrates) exceeding the growth requirements of *C. pilosula*, and some primary metabolites could be converted into secondary metabolites to effectively avoid wasting resources (Gong et al., 2022). In addition, active ingredient yield was positively correlated with leaf net photosynthetic rate and C metabolism of *C. pilosula*, and PLS-PM showed that leaf size and net photosynthetic rate can indirectly promote active ingredient concentration by increasing C and N metabolism. This further supported the growth-differentiation balance hypothesis. However, how intercropping affects the secondary metabolic processes of *C. pilosula* is unclear and needs further study.

## 5 Conclusion

Our study demonstrated that appropriate configuration of *C. pilosula*/faba bean intercropping leads to the dual promotion of root yield and active ingredients concentration of *C. pilosula*, resulting in a higher accumulation of active ingredients. Those results could be attributed to improved light environment, photosynthesis as well as C metabolism of leaves in intercropping systems. Our findings highlighted the importance of aboveground interaction and helped us to better understand the overyielding mechanisms underlying medicinal-based intercropping systems. However, the lack of data for faba bean limited a deeper understanding of the interspecific interaction, which needs to be considered in the future. Our study suggested that two rows of *C. pilosula* intercropping with one row of faba bean is a promising planting pattern for the ecological cultivation of *C. pilosula*, which is vital to the sustainable production of traditional Chinese medicine.

## Data Availability

The original contributions presented in the study are included in the article/supplementary material. Further inquiries can be directed to the corresponding authors.
